# The role of glia in epilepsy, intellectual disability, and other neurodevelopmental disorders in tuberous sclerosis complex

**DOI:** 10.1186/s11689-019-9289-6

**Published:** 2019-12-16

**Authors:** Michael Wong

**Affiliations:** Department of Neurology and the Hope Center for Neurological Disorders, Washington University School of Medicine, 660 South Euclid Avenue, Box 8111, St. Louis, MO 63110 USA

**Keywords:** TAND, Intellectual disability, Autism spectrum disorder, Epilepsy, Tuberous sclerosis, Glia, Astrocyte, Microglia, Oligodendrocyte, White matter

## Abstract

**Background:**

Tuberous sclerosis complex (TSC) is a genetic disorder characterized by severe neurological manifestations, including epilepsy, intellectual disability, autism, and a range of other behavioral and psychiatric symptoms, collectively referred to as TSC-associated neuropsychiatric disorders (TAND). Various tumors and hamartomas affecting different organs are the pathological hallmarks of the disease, especially cortical tubers of the brain, but specific cellular and molecular abnormalities, such as involving the mechanistic target of rapamycin (mTOR) pathway, have been identified that also cause or contribute to neurological manifestations of TSC independent of gross structural lesions. In particular, while neurons are immediate mediators of neurological symptoms, different types of glial cells have been increasingly recognized to play important roles in the phenotypes of TSC.

**Main body:**

This review summarizes the literature supporting glial dysfunction from both mouse models and clinical studies of TSC. In particular, evidence for the role of astrocytes, microglia, and oligodendrocytes in the pathophysiology of epilepsy and TAND in TSC is analyzed. Therapeutic implications of targeting glia cells in developing novel treatments for the neurological manifestations of TSC are also considered.

**Conclusions:**

Different types of glial cells have both cell autonomous effects and interactions with neurons and other cells that are involved in the pathophysiology of the neurological phenotype of TSC. Targeting glial-mediated mechanisms may represent a novel therapeutic approach for epilepsy and TAND in TSC patients.

## Background

Tuberous sclerosis complex (TSC) is one of the classic neurocutaneous syndromes, featuring characteristic pathological brain and skin lesions, as well as tumors in a number of other organs [1, 2]. With brain involvement, TSC is often characterized by a severe neurodevelopmental disorder, aptly named TSC-associated neuropsychiatric disorders (TAND), including intellectual disability, autism, and other behavioral and psychiatric symptoms [3, 4]. Some degree of cognitive dysfunction, ranging from mild learning disabilities to severe intellectual disability, affects at least 50% of TSC patients. Similarly, autism spectrum disorder or other behavioral disorders also occur in about half of TSC patients. In addition, epilepsy is extremely common affecting up to 80% of TSC patients, with seizures usually being severe and intractable to treatment and often exacerbating the cognitive and behavioral comorbidities [5].

TSC is caused by mutations in one of two genes, the *TSC1* and *TSC2* genes [1, 2]. These genes encode for two proteins, hamartin (TSC1) and tuberin (TSC2), which bind together to form a protein dimer complex that inhibits the mechanistic target of rapamycin (mTOR) pathway. mTOR is a protein kinase, which serves as a central regulator of a number of important physiological functions, such as cell growth and proliferation, metabolism, and protein synthesis [6, 7]. In TSC, mutation of *TSC1* or *TSC2* leads to a disinhibition or hyperactivation of the mTOR pathway, which promotes increased cell growth and proliferation and tumor formation. This cellular growth dysregulation leads to the variety of tumors seen in TSC, including subependymal giant cell astrocytomas (SEGA) in the ventricles of the brain, renal angiomyolipomas of the kidneys, lymphangioleiomyomatosis in the lungs, and facial angiofibromas of the skin. mTOR inhibitors are now FDA approved treatments for these brain, kidney, and lung tumors in TSC [8–10] and is also effective against the facial angiofibromas [11]. While mTOR inhibitors, such as rapamycin or everolimus, are clearly effective against different tumor types in TSC, their efficacy against neurological symptoms of TSC is more limited. Adjunctive treatment with everolimus has been shown to have efficacy for focal seizures in TSC patients with drug-resistant epilepsy [12, 13], but the majority of TSC patients continue to have seizures (i.e., do not become seizure-free) and many patients showed minimal benefit from treatment. Furthermore, everolimus was found to have no efficacy against TAND in a battery of neurocognitive and behavioral tests in one recent placebo controlled-trial [14]. Thus, more effective treatments are needed for both TAND and epilepsy in TSC.

**Fig. 1:**
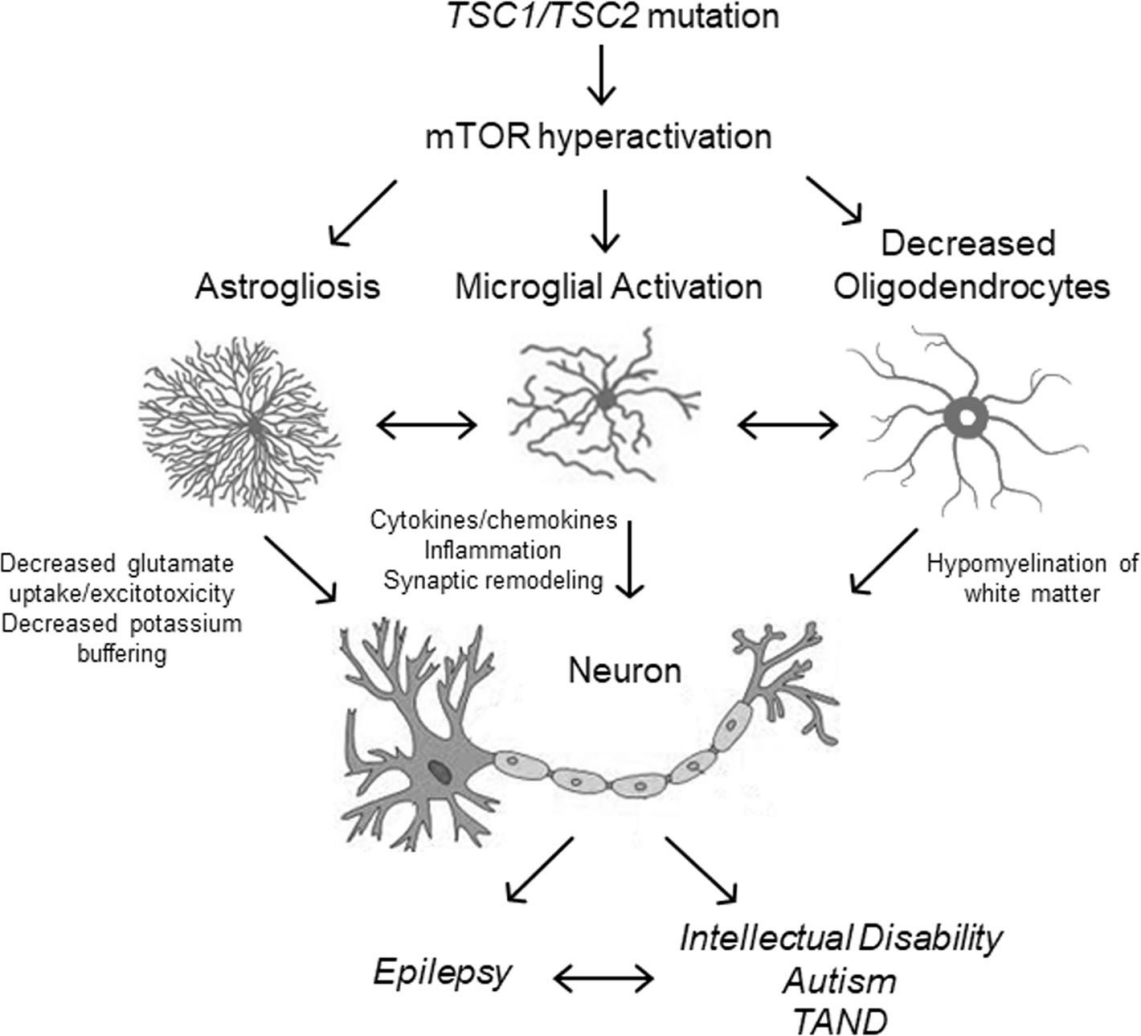
Schematic overview of the potential role of glia in the neurological phenotypes of TSC. *TSC1* or *TSC2* gene mutations lead to abnormal hyperactivation of the mechanistic target of rapamycin (mTOR) pathway, which can directly (through cell autonomous effects) or indirectly (through interactions with other cells) cause astrogliosis, microglial activation, and decreased oligodendrocytes. These glial abnormalities can then affect neuronal function through multiple mechanisms, such as impaired glutamate and potassium homeostasis, synaptic remodeling, inflammatory processes, and hypomyelination, which ultimately lead to epilepsy, intellectual disability, autism, and other TSC-associated neuropsychiatric disorders (TAND)

Compared with the mechanisms of tumorigenesis in TSC, the pathophysiology of TAND and epilepsy in TSC is poorly understood. Independent of the SEGAs, the classic pathological brain lesion in TSC is the cortical tuber, which gives the disease its name, based on the potato-like appearance on gross pathology. Unlike SEGAs, cortical tubers are focal malformations of cortical development, consisting of localized areas of disrupted cortical lamination and a variety of cellular abnormalities, including astrogliosis, dysmorphic neurons, and giant cells, which are enlarged undifferentiated cells with immature glial and neuronal markers. Cortical tubers are traditionally thought to cause or contribute to neurological manifestations of TSC. There is a correlation between the number of tubers or “tuber load” and the severity of intellectual disability [15]. Furthermore, some studies suggest that the risk of autism may relate to tubers localized to the temporal lobes [16]. However, the correlation between tubers and TAND is non-specific and controversial, not being demonstrated in all studies [17, 18]. There is increasing evidence that cognitive dysfunction and autism are more directly related to tuber-independent abnormalities in the brain, such as disrupted functional connectivity of white matter. There is stronger evidence that epilepsy may be caused by tubers, at least in some cases, as surgical removal of tubers can sometimes eliminate seizures in some TSC patients [19]. However, even when tubers cause seizures, it is still controversial as to whether the seizures start within the tubers themselves or in the surrounding perituberal region [20, 21]. Regardless of whether seizures start in, around, or independent of tubers, there is increasing evidence that dysregulated cellular and molecular processes also drive epileptogenesis [22]. On the cellular level, while neurons clearly are centrally involved in the brain phenotype of TSC, an attractive novel hypothesis is that abnormalities in glial cells may contribute to the neurological manifestations of TSC (Fig. 1). In this review, we will examine the evidence for different types of glial abnormalities in TSC and their potential role in promoting TAND and epilepsy in TSC.

## Main text

### Astrocytes

While traditionally astrocytes have been viewed as passive, supportive cells for neurons in the brain, the modern concept of astrocytes entails a more active role in a variety of brain functions [23]. The list of physiological functions of astrocytes continues to grow, including metabolism, structural support, blood-brain barrier maintenance, neurotransmitter regulation and turnover, and direct intercellular communication with other astrocytes and neurons (“gliotransmission”). Astrocytes are critical for maintaining proper energetic balance within the brain, supplying lactate and other nutrients to neurons. Astrocytic processes and endfeet form a component of the blood-brain barrier in conjunction with endothelial cells of the cerebral vasculature. Neurotransmitter transporters on astrocytes, such as for glutamate, absorb glutamate released into synapses by neurons, helping to terminate the synaptic signal and to prevent excitotoxicity. Similarly, astrocytes regulate extracellular potassium homeostasis, which affects neuronal excitability. Perhaps most emblematic of the active role of astrocytes in brain physiology, astrocytes can release gliotransmitters and participate directly in cellular signaling with other astrocytes and neurons through gliotransmission. The diversity of astrocyte functions is paralleled by the heterogeneity of astrocytes, including at least protoplasmic and fibrillary subtypes [24]. Corresponding to the variety of physiological functions in the brain, astrocyte dysfunction can potentially contribute to the pathophysiology of neurological disorders.

The role of astrocytes in TSC was first implicated in pathological brain specimens from TSC patients. Astrogliosis as generally reflected by a change in morphology and increased glial-fibrillary acidic protein (GFAP) staining is a prominent feature of cortical tubers [25–27]. At least two types of morphologically abnormal astrocytes have been described within tubers: “gliotic” astrocytes with elongated radial processes and abundant intermediate filaments and occasional “reactive” astrocytes with increased cell size and vimentin expression often adjacent to giant cells [27]. The cause of astrogliosis in TSC is not known, but could be a primary cell-autonomous effect of *TSC* gene inactivation directly within astrocytes or result secondarily from neuronal abnormalities or seizures that indirectly affect astrocytes. Supporting the former possibility, biochemical evidence of mTOR activation can be detected at least within reactive astrocytes, indicating that the increased cell size is directly caused by *TSC* gene mutation and resulting mTOR hyperactivity [27].

As it is difficult to determine the functional effects of astrocyte abnormalities in human studies, animal models of TSC can more directly examine the role of astrocytes in the pathophysiology of the neurological manifestations of TSC. A variety of knockout or transgenic mouse models of TSC target the brain, involving *Tsc1* or *Tsc2* gene inactivation in different cell types, including both neurons and glia (Table 1). The TSC mouse model that has most extensively investigated astrocytic mechanisms is a conditional knockout mouse, *Tsc1*^GFAP^CKO mice, in which a glial fibrillary acidic protein (GFAP) promoter has been used to drive *Tsc1* gene inactivation in astrocytes [28]. On a behavioral level, these mice exhibit cognitive deficits in spatial learning [29], as well as severe epilepsy [30]; thus, *Tsc1*^GFAP^CKO mice appear to recapitulate some of the key neurological manifestations of TSC.

**Table 1:** Mouse models of TSC targeting glia

		Cognitive			
Mouse Model	Glial pathology	Epilepsy	Deficits	Other/comment	References
Astrocytes					
Gfap-*Tsc1* CKO mice	Astrogliosis/proliferation Increased astrocyte size Impaired glutamate transport, potassium channels, and gap junctions.	+	+	Not specific for astrocytes, some neuronal involvement	[28–36]
Gfap-*Tsc2* CKO mice	Astrogliosis/proliferation Increased astrocyte size Impaired glutamate transport	+	n/a	Not specific for astrocytes, some neuronal involvement	[37]
Gfap2-*Tsc1* CKO mice	Astrogliosis/proliferation Decreased myelination	+	n/a	Not specific for astrocytes, some neuronal involvement	[38]
Gfap2-*Tsc2* CKO mice	Astrogliosis/proliferation Decreased myelination	+	n/a	Not specific for astrocytes, some neuronal involvement	[39, 40]
Inducible GFAP-*Tsc1* CKO mice	Astrogliosis/proliferation	+/−	n/a	Postnatal *Tsc1* inactivation more specific for astrocytes	[41]
Microglia					
Cx3cr1-*Tsc1* CKO mice	Microglia activation Increased microglia size and number.	+	n/a	Controversial as to specificity for microglia	[42, 43]
Inducible Cxcr1-*Tsc1* CKO mice	Microglia activation Increased microglia size and number	+/−	n/a	Postnatal *Tsc1* inactivation more specific for microglia	[42, 43]
Oligodendrocytes					
Olig2-*Tsc2* CKO mice	Hypomyelination Decreased oligodendrocyte number	–	–		[44]

*n/a* not reported

A number of structural and functional abnormalities have been identified in astrocytes that may contribute to the cognitive deficits and epilepsy in *Tsc1*^GFAP^CKO mice. On the pathological level, *Tsc1*^GFAP^CKO mice exhibit widespread astrocyte proliferation due to mTOR hyperactivation, leading to diffuse megalencephaly of the brain [28, 31]. Also directly related to mTOR activation, astrocyte cell size is increased in the *Tsc1*^GFAP^CKO mice [32]. The specific functional consequences of the increased astrocyte proliferation and size are not entirely clear, but it is reasonable to hypothesize that the gross megalencephaly and disruption of neuronal networks from astroproliferation and astrogliosis could adversely affect neuronal function and excitability, leading to the behavioral deficits and seizures.

In addition to these histological and morphological abnormalities, *Tsc1*-knock out astrocytes exhibit a number of molecular defects that interfere with their functional properties. Astrocytes normally contain glutamate transporters, such as glutamate transporter 1 (Glt-1), which remove glutamate from synapses and terminate the synaptic signal. *Tsc1*^GFAP^CKO mice have decreased Glt-1 expression and a corresponding reduction in astrocyte glutamate transporter function [33], which leads to elevated extracellular glutamate levels and excitotoxic neuronal death [29]. Somewhat paradoxically, excessive synaptic glutamate results in impaired synaptic plasticity of long-term potentiation (LTP), a mechanism of learning and memory [29]. Increased glutamate may logically also promote neuronal hyperexcitability that causes seizures. Thus, astrocyte dysfunction related to glutamate homeostasis may lead to the behavioral learning deficits and epilepsy seen in *Tsc1*^GFAP^CKO mice.

A number of other astrocyte defects have been found in *Tsc1*^GFAP^CKO mice, including decreased potassium channel functioning and impaired gap junctions. Similar to glutamate uptake, astrocytes normally play a significant role in buffering extracellular potassium through inward-rectifying potassium channels, which absorb potassium. Networks of astrocytes communicate with each other through gap junctions, which allow redistribution and further buffering of potassium. *Tsc1*^GFAP^CKO mice have decreased potassium channel expression and a reduction in potassium buffering capability [34, 35]. The decreased potassium buffering by *Tsc1* KO astrocytes leads to neuronal hyperexcitability, which may promote seizures and cognitive dysfunction.

If astrocyte abnormalities are necessary for the neurological manifestations, treatments that reverse these abnormalities should prevent or improve the neurological symptoms. Ceftriaxone, an antibiotic drug that also increase astrocyte glutamate transporter expression, can reduce seizures in *Tsc1*^GFAP^CKO mice, providing evidence that impaired astrocyte glutamate transport contributes to the epilepsy phenotype [36]. Furthermore, the mTOR inhibitor, rapamycin reverses the astrocyte proliferation and associated megalencephaly in *Tsc1*^GFAP^CKO mice and can prevent epilepsy in these mice. Given that GFAP is also expressed in neuroprogenitor cells, the simultaneous contribution of neuronal abnormalities is difficult to rule out in *Tsc1*^GFAP^CKO mice and the effect of specific *Tsc1* inactivation in astrocytes independent of neurons is more limited [41]. However, overall, these studies suggest that astrocyte abnormalities contribute to epileptogenesis and cognitive dysfunction in *Tsc1*^GFAP^CKO mice and support novel treatment approaches for neurological manifestations of TSC targeting astrocytes.

### Microglia

Microglia represent the resident macrophages of the central nervous system, primarily mediating innate and adaptive immune responses in the brain, such as in reaction to CNS infections, neurodegenerative diseases, or other brain injury [45]. Microglia differ from other glia in originating outside of the brain from myeloid, rather than neuroectoderm, progenitors, and migrating into the brain during embryogenesis. Microglia may exist in two morphologically and functionally distinct states: a resting and activated state. In their activated state, microglia function to clear cellular debris and produce cytokines and chemokines which coordinate other cellular immune responses from astrocytes, neurons, and lymphocytes. In addition to their central role in immune responses in the brain, microglia have also been found to modulate brain development by regulating neurogenesis, neuronal migration, and synaptic maturation, wiring, and pruning [45]. In contrast to the beneficial functions of microglia, in disease states microglia activation may contribute to pathological processes that are detrimental to the brain.

In TSC, there is pathological evidence of microglial activation within tubers from TSC patients [46, 47]. In cortical tuber specimens resected from TSC patients undergoing epilepsy surgery for intractable epilepsy, prominent activated microglia are identified based on their characteristic morphology and positive staining for markers of microglia activation, HLA-DR and CD68. Microglia are often clustered around dysmorphic neurons and giant cells and are also associated with other immune mediators, including CD8-positive T lymphocytes and components of the complement cascade. These pathological findings suggest that microglia can play a role in the pathophysiology of neurological manifestations of TSC. However, as seizures themselves may cause microglia activation [48], it is difficult to determine whether the microglia activation in tuber specimens from TSC patients with epilepsy is a primary pathophysiological mechanism or is simply secondary to seizures.

Although it is not clear to what degree *TSC* gene inactivation and associated mTOR hyperactivation occur directly in microglia in the human pathological studies, targeted *Tsc* gene inactivation in mouse models can help address the question of whether microglia abnormalities may play a primary role in the pathogenesis of TSC. First of all, *Tsc1*^GFAP^CKO mice exhibit elevated Iba1 staining, a marker of microglia activation, and increased microglia size and number [49]. Minocycline, a drug that can inhibit microglia activation, is able to prevent the morphological changes in microglia, but has no effect on seizures, suggesting that the microglia activation is a secondary effect that does not cause epilepsy in *Tsc1*^GFAP^CKO mice. This result is perhaps not surprising, given that GFAP-driven *Tsc1* inactivation is expected to affect astrocytes and neurons, but not microglia directly.

Recent studies have attempted to inactivate *Tsc* genes directly in microglia, such as using a Cx3 chemokine receptor 1 (Cx3cr1) driver, which is a chemokine receptor that is traditionally thought to be specifically expressed in microglia. *Tsc1*^Cx3Cr1^CKO mice exhibit mTOR hyperactivation in microglia and resulting increased microglia size and number, indicating that *Tsc1*inactivation has cell autonomous effects in microglia [42, 43]. *Tsc1*^Cx3Cr1^CKO mice have severe epilepsy, as well as megalencephaly, reduced synaptic density, and neuronal degeneration, although a neurocognitive or behavioral phenotype has not been reported. This suggests that intrinsic microglia abnormalities may be sufficient to at least cause epilepsy in TSC. However, there is some controversy as to the specificity of *Tsc1* inactivation in *Tsc1*^Cx3Cr1^CKO mice, which may not be limited to microglia but likely also affects neurons, and whether more specific postnatal *Tsc1* inactivation in microglia causes epilepsy [42]. Thus, microglia may contribute to or modulate the neurological manifestations of TSC, but might also require concurrent neuronal abnormalities. Future animal model studies with more selective targeting of microglia may help resolve the specific role of microglia in epilepsy in TSC more definitively, as well as examine their effects on cognitive function.

### Oligodendrocytes

Oligodendrocytes are the third major type of glia cell in the central nervous system and are most directly involved in development and maintenance of the white matter of the brain [50]. Akin to Schwann cells in the peripheral nervous system, the primary function of oligodendrocytes is to form the myelin insulation of axons, allowing for efficient and rapid conduction of action potential signaling along white matter tracts between brain regions. Oligodendrocytes and myelin exhibit significant heterogeneity and are not uniformly distributed throughout the brain, suggesting that oligodendrocytes play a differential role in regulating brain function and neuronal networks [51, 52]. Dysfunction or degeneration of oligodendrocytes are the cardinal feature of demyelinating or dysmyelinating diseases, such as multiple sclerosis, which often feature cognitive impairment, in addition to more classic focal neurological deficits.

Although cortical tubers have classically been the pathological hallmark of the neurological phenotype of TSC, white matter abnormalities have emerged as an equally important and distinctive mechanism for brain dysfunction in TSC. Pathological studies have found decreased myelin content and oligodendrocyte number in and around cortical tuber specimens [53]. This decrease in myelin and oligodendrocytes within tubers has been linked to a deficiency in oligodendrocyte progenitor cells and elevated mTOR activity [53], suggesting that TSC involves a primary defect in oligodendrocytes related to *TSC* gene inactivation.

A plethora of MRI studies have further documented abnormalities in white matter that are much more extensive and diffuse than just tubers. In particular, diffusion tensor imaging (DTI) has been used to evaluate microstructural changes in white matter, based on the general principle that water diffusion in normal white matter is directionally restricted primarily to parallel to the orientation of axons (anisotropy), whereas disruption of the normal organization of white matter leads to increased diffusion in other directions. Multiple MRI studies using DTI have documented increased mean diffusivity and decreased anisotropy in white matter of TSC patients in the corpus callosum, subcortical white matter, internal capsule, and other white matter tracts that appear grossly normal on MRI and are remote from tubers [54–56], indicating disruption in microstructural organization and abnormal myelination of white matter in TSC.

In terms of the functional significance of these white matter abnormalities, TSC patients with autism spectrum disorder have more severe abnormalities in DTI parameters compared with TSC patients without ASD and control patients, whereas there is no significant difference between TSC patient without ASD and controls [57]. When examining white matter pathways involved in language processing, particularly the arcuate fasciculus, TSC patients with ASD have abnormalities in diffusivity and anisotropy compared with TSC patients without ASD, although there are also additional differences between TSC patients without ASD and controls [58]. These white matter abnormalities are associated with an overall decrease in measures of functional connectivity between different regions of the brain, including reduced interhemispheric synchrony [59]. Furthermore, the degree of white matter abnormalities is also correlated with the presence of seizures [60]. Overall, epilepsy, intellectual disability, and ASD individually seem to have additive effects on the abnormal DTI measures [61].

From a therapeutic standpoint, an important question is whether these white matter abnormalities may be reversible. Interestingly, the mTOR inhibitor everolimus produces decreases in diffusivity and increases in anisotropy in serial DTI studies of TSC patients [62]. Longitudinal studies have found that longer periods of treatment with everolimus results in greater effects [63]. The mechanism of this effect of everolimus on these DTI parameters is not known, but could be related directly to structural or metabolic effects on oligodendrocytes or axons, such as decreases in extracellular fluid or cellular volume and improved myelination or myelin leakiness, or indirectly to a decrease in seizures. In any case, these exciting findings suggest that white matter abnormalities in TSC can be reversed by treatment, which provides a potential mechanistic avenue for therapeutic interventions for cognitive dysfunction and ASD in TSC patients.

Animal models and other reduced systems have delved further into the mechanistic basis of white matter abnormalities in TSC, which could result either from cell autonomous effects of *TSC* gene inactivation in oligodendrocytes or abnormal signaling from TSC-deficient neurons or astrocytes that indirectly affect oligodendrocytes. Inactivation of *Tsc2* directly in oligodendrocytes in mice using a Olig2 promoter leads to a marked hypomyelination phenotype, supporting a cell autonomous effect of oligodendrocytes directly [44]. This hypomyelination is related to decreased oligodendrocyte number from a shift in oligodendrocyte precursor differentiation from oligodendrocytes to astrocytes, as well as to decreased myelin thickness. In addition, mice with neuron-specific inactivation of *Tsc1* also demonstrate a hypomyelination phenotype, supporting that abnormal communication from neurons to oligodendrocytes can cause white matter abnormalities [64]. This neuronal regulation of oligodendrocyte-mediated myelination is mediated by connective tissue growth factor secreted by neurons, which then negatively regulates oligodendrocyte development [65]. Conversely, TSC-deficient oligodendrocytes, derived from human induced pleuripotent stem cells (iPSCs) from TSC patients, can affect the morphological and physiological properties of neurons, suggesting a bi-directional regulation between oligodendrocytes and neurons [66]. So, overall, there is evidence of both cell-autonomous effects of oligodendrocytes and interactions between neurons and oligodendrocytes in causing white matter abnormalities in TSC.

## Conclusions and future directions

Glia cells of different types have emerged as major players in causing or contributing to TAND and other neurological phenotypes of the genetic disorder, TSC. While neurons remain the cardinal cell of the brain directly mediating neurological manifestations, both cell autonomous actions of glia and interactions of glia and neurons appear critical for a variety of brain symptoms of TSC, including intellectual disability, autism, epilepsy, and other psychiatric and behavioral disorders. However, there are a number of outstanding issues that need to be resolved in further defining the role of glia in TSC.

As neurons and glia work together in complex, interdependent networks, it is difficult to isolate and disentangle the relative contribution and role of glia in neurological manifestations. Knockout mice specifically targeting glia cells have clearly established cell autonomous effects of *Tsc* gene inactivation directly within glia. However, proving that these effects by themselves are sufficient to cause neurological manifestations or are co-dependent on *Tsc* gene inactivation in neurons has been difficult. In addition, it is not clearly resolved whether germline mutation of a single TSC allele in the heterozygous state is sufficient to cause neurological symptoms or a “second hit” involving an additional somatic mutation and resulting in a homozygous mutant state is required, especially in the human disease. Simplified systems, such as induced pleuripotent stem cell (iPSC)-derived neurons and glia, can be utilized to better address these questions of cell-autonomous versus interdependent effects and heterozygous versus homozygous states.

Another long-standing controversy within the TSC field is the role of tubers in causing neurological manifestations. Tubers have traditionally been thought of as being the critical pathological substrate, with tubers directly causing seizures and with tuber load correlating with intellectual disability and autism. However, microstructural defects in non-tuber parts of the brain have received increasing attention as contributing to the neurological phenotype of TSC. Glial cells, in particular, provide an obvious cellular platform for mediating brain dysfunction independent of gross structural lesions, such as in the case of oligodendrocytes and white matter abnormalities in TSC. Ultimately, there may be a continuum of glial defects between tubers, perituberal regions, and remote structurally “normal” areas of the brain. Increasingly sophisticated imaging studies examining brain connectivity and correlating with pathological and neurophysiological parameters can further determine the interrelationship between tuber and non-tuber areas of the brain in causing neurological manifestations of TSC in general, as well as in relation to glia.

The wide range of neurological symptoms of TSC, encompassed by the term TAND, as well as epilepsy, raises mechanistic and therapeutic questions as to the causal or correlative relationship between different symptoms. In particular, do overlapping networks and cellular elements cause diverse neurological manifestations or are there independent, distinctive mechanisms for each symptom? Furthermore, do some manifestations, particularly seizures, directly exacerbate other symptoms, such as intellectual disability? Again, omnipresent, highly interconnected glial cells throughout the brain represent a natural substrate for mediating interactions between the different neurological phenotypes of TSC. Understanding and targeting these overlapping glial features may provide opportunities for therapeutic interventions that simultaneously and synergistically benefit multiple neurological manifestations of TSC.

Finally, glial cells do represent a potential novel therapeutic target for neurological symptoms of TSC. Current treatments for epilepsy primarily regulate neuronal mechanisms, such as by directly controlling neuronal excitability via modulation of ion channels or neurotransmitter systems, and there are essentially no specific pharmacological treatments for most of the manifestations of TAND. One of the major limitations of current neuroactive medications is sedation and cognitive slowing due to depression of normal neuronal activity. Targeting glial cells has the potential to modulate neuronal networks without directly causing neuronal depression. While the emergence of mTOR inhibitors as a treatment for TSC has revolutionized the targeted therapeutic approach to TSC in general, limitations in efficacy for neurological symptoms of TSC and systemic side effects of mTOR inhibitors indicate that additional directed strategies to treating neurological manifestations of TSC. Given the prevalence of glial abnormalities in TSC, TSC has the potential to be a model disease for investigating and targeting glia as novel therapeutic approaches to neurodevelopmental disorders in general.

## Data Availability

n/a

## Abbreviations

ASD: Autism spectrum disorder
Cx3cr1: Cx3 chemokine receptor 1
DTI: Diffusion tensor imaging
FDA: Food and Drug Administration
GFAP: Glial fibrillary acidic protein
Glt-1: Glutamate transporter 1
iPSCs: Induced pleuripotent stem cells
LTP: Long-term potentiation
mTOR: Mechanistic target of rapamycin
SEGA: Subependymal giant cell astrocytoma
TAND: TSC-associated neuropsychiatric disorders
TSC: Tuberous sclerosis complex
*Tsc1*^GFAP^CKO mice: *Tsc1*-GFAP conditional knockout mice
